# Health education website on home care for newborns: construction, validation, and evaluation ^*^

**DOI:** 10.1590/1518-8345.7222.4197

**Published:** 2024-06-17

**Authors:** Iasmym Alves de Andrade Soares, Fernanda Garcia Bezerra Góes, Aline Cerqueira Santos Santana da Silva, Fernanda Maria Vieira Pereira-Ávila, Gabrielle Beltrão de Oliveira, Maria da Anunciação Silva

**Affiliations:** 1 Universidade Federal Fluminense, Instituto de Humanidades e Saúde, Rio das Ostras, RJ, Brazil.; 2 Universidade Federal do Estado do Rio de Janeiro, Rio de Janeiro, RJ, Brazil.; 3 Scholarship holder at the Fundação Carlos Chagas Filho de Amparo à Pesquisa do Estado do Rio de Janeiro (FAPERJ), Brazil.

**Keywords:** Educational Technology, Computer Communication Networks, Family, Postnatal Care, Infant Newborn, Health Education, Tecnología Educacional, Redes de Comunicación de Computadores, Familia, Atención Posnatal, Recién Nacido, Educación en Salud, Tecnologia Educacional, Redes de Comunicação de Computadores, Família, Cuidado Pós-Natal, Recém-Nascido, Educação em Saúde

## Abstract

**Objective:**

to build, validate, and evaluate an educational health website on home care for newborns for use by pregnant women, postpartum women, and family members.

**Method:**

methodological study developed according to the Analyze, Design, Develop, Implement, and Evaluate model. After construction, the website was validated by 20 experts and evaluated by 20 individuals from the target audience, and the data wasanalyzed according to the Concordance Index with a cut-off point equal to or greater than 0.7 (70%).

**Results:**

in the validation, the Concordance Index for all the items was higher than 0.7 (70%), with a variation between 0.75 (75%) and 1 (100%), reaching an overall average value of 0.91 (91%). In the evaluation, all the items got top marks, with anoverall average value of 1 (100%).

**Conclusion:**

the educational website was built, validated, and evaluated in a satisfactory manner. It can be considered an appropriate tool for its purpose, with benefits in the teaching-learning process for families regarding postnatal home care fornewborns through its use. It can also be used to educate students and health professionals. The website is available for free access via laptops, computers, smartphones, or tablets.

## Abstract (es)

**Objetivo:**

construir, validar y evaluar un sitio *web* educativo en salud sobre cuidados domiciliarios del recién nacido para uso de mujeres embarazadas, puérperas y sus familias.

**Método:**

estudio metodológico desarrollado según el modelo *Analyze, Design, Develop, Implement and Evaluate* . Luego de su construcción, el sitio *web* fue validado por 20 expertos y evaluado por 20 personas del público objetivo, y los datos analizados según el Índice de Concordancia con un punto de corte igual o superior a 0,7 (70%).

**Resultados:**

en la validación, el Índice de Concordancia para todas las cuestiones fue superior a 0,7 (70%), con una variación entre 0,75 (75%) y 1 (100%), alcanzando un valor promedio general de 0,91 (91%). En la evaluación, todos los ítems obtuvieron la máxima puntuación, con un valor medio global de 1 (100%).

**Conclusión:**

el sitio *web* educativo fue construido, validado y evaluado de manera satisfactoria y puede ser considerado una herramienta adecuada para su objetivo, aportando beneficios al proceso de enseñanza-aprendizaje de las familias sobre el cuidado domiciliario posparto del recién nacido por medio de su uso. Además, también se puede aplicar a la educación de estudiantes y profesionales de la salud. El sitio *web* está disponible para acceso libre y gratuito por medio de *notebooks* , computadoras, *smartphones* o *tablets* .

## Abstract (pt)

**Objetivo:**

construir, validar e avaliar um *website* educacional em saúde sobre os cuidados domiciliares com os recém-nascidos para uso de gestantes, puérperas e familiares.

**Método:**

estudo metodológico desenvolvido conforme o modelo *Analyze, Design, Develop, Implement and Evaluate* . Após a construção, o *website* foi validado por 20 *experts* e avaliado por 20 indivíduos do público-alvo e os dados analisados conforme Índice de Concordância com ponto de corte igual ou maior a 0,7 (70%).

**Resultados:**

na validação, o Índice de Concordância de todos os quesitos foi maior do que 0,7 (70%), com variação entre 0,75 (75%) e 1 (100%), alcançando valor médio global de 0,91 (91%). Na avaliação, todos os itens obtiveram nota máxima, com valor médio global de 1 (100%).

**Conclusão:**

o *website* educacional foi construído, validado e avaliado de maneira satisfatória, pode ser considerado uma ferramenta adequada ao seu objetivo, com benefícios no processo de ensino-aprendizagem das famílias, quanto ao cuidado pós-natal domiciliar com os recém-nascidos mediante sua utilização. Além disso, também pode ser aplicada na educação de estudantes e profissionais de saúde. O *website* encontra-se disponível para o acesso livre e gratuito por meio de *notebooks* , computadores, *smartphones* ou *tablets* .

## Introduction

 Over the last few decades, the world community has sought to reduce neonatal mortality. However, every year, 2.5 million newborns still die in the first month of life, and almost three-quarters of these deaths occur in the first week after birth ^(^ 1 ^)^ , mainly for preventable reasons ^(^ 2 ^)^ . Many die in the socio-familial context because of the short length of stay in hospital after childbirth, a circumstance that could be avoided by providing guidance to families, from prenatal to puerperium, on practices that have been proven to reduce illnesses and deaths, such as: maintaining the baby’s body temperature; early and exclusive breastfeeding; preventing infections; timely recognition of illnesses and seeking professional care in case of need ^(^ 1 ^)^ . 

 A study that analyzed the learning demands of puerperal women and family members regarding postnatal care for newborns identified that there are demands to be met, mainly related to the baby’s body hygiene, including bathing and umbilical stump care, as well as issues such as nutrition and the use of artificial nipples ^(^ 3 ^)^ . Another study also identified the various learning needs of families of premature babies in intensive care. It was found that their information needs are wide-ranging since they have questions about the development of their newborns, prognosis, and survival after hospital discharge, as well as post-natal care, carried out at home, such as administering milk supplements. It was also found that these families use a variety of means to find answers, including the use of websites ^(^ 4 ^)^ . 

 Evidence shows that newborn care is an aspect of health that is more likely to be improved with counseling, which suggests that health education can be an effective way to help improve the baby’s healthy development outcomes ^(^ 1 ^)^ . In this way, health professionals, including nurses, should promote educational actions in order to minimize the challenges of the neonatal period, such as the anxiety of family members and the vulnerability of the newborn, contributing to better child growth and development ^(^ 5 ^)^ . 

 The association of health care coordination with technological tools based on digital health, in order to promote hybrid care (face-to-face and virtual), is necessary, especially with the advent of Coronavirus Disease (COVID-19) ^(^ 6 ^)^ , and has been considered institutionally relevant in Brazil, especially after the publication of the Brazilian Digital Health Strategy (2020-2028), an innovation for the Unified Health System (UHS) ^(^ 7 ^)^ . In this sense, educational technologies in health are of great importance, as they are instruments that facilitate teaching and learning and enhance health education ^(^ 8 ^)^ . They are characterized by the participation of the individual in a moment of exchange of experiences and improvement of skills, being a creative and attractive way of disseminating knowledge that will bring benefits to users and also an optimization of the professional’s work, streamlining the educational process ^(^ 9 ^)^ . Therefore, they seek to improve the quality of life of the population involved through the construction of various technological products, such as protocols, digital applications, and folders, among others ^(^ 10 ^)^ . 

 Among these possibilities are websites, and virtual pages with various technological resources that generate low costs and greater reach and motivation among the public ^(^ 11 ^)^ . Websites are accessible, available regardless of location or time, and allow for user anonymity ^(^ 12 ^)^ . In addition, they can include personalized information and reach a larger group of people than face-to-face interventions ^(^ 13 ^)^ . For this reason, the use of innovative strategies in communicating with the target audience, such as the website, makes it possible to capture the main demands of individuals, valuing their knowledge and sensitizing health professionals to the best care practices, that are safe and of quality ^(^ 5 ^)^ . 

 Given this, an educational health website is an excellent tool for communicating and disseminating scientific information, presenting itself as a fast means of informing, sharing, and exchanging experiences, given the integration of different information on the same topic ^(^ 14 ^)^ . 

Thus, the desire arose to bring together content related to home care for newborns, hosting it in a clear and didactic way on an educational health website. The development of the website is classified as an innovative proposal, which aims to provide pregnant women, puerperal women, and family members with tools on post-natal home care for babies, in order to meet their learning demands and contribute to best practices, preventing illnesses and reducing infant morbidity and mortality. In addition, literature searches were carried out on websites related to post-natal care for newborns, and no studies of this nature were found in the Brazilian context, which justifies this study.

The aim was to build, validate, and evaluate a health education website on home care for newborns for use by pregnant women, postpartum women, and family members.

## Method

### Type of study

 This is a methodological study of the construction, validation, and evaluation of health educational technology in website format according to the Analyze, Design, Develop, Implement, and Evaluate (ADDIE) model, a classic Instructional System Design flow containing five stages ^(^ 15 ^)^ . 

 The first stage, Analysis, involves identifying the problems encountered that led to the construction of the educational technology, as well as defining the goals and objectives of the construction, taking into account the expectation of the technology’s instructional objective ^(^ 15 ^)^ . Thus, the intention to build, validate, and evaluate the website came from the experience and previous contact with the theme of home care for newborns in various contexts, i.e. as an extension and research project, scientific initiation, technological initiation, and practical teaching field, a trajectory that pointed to the need for an educational technology in health in the format of a website that brings together in a single place the various thematic contents and educational materials already built and validated. Therefore, the instructional objectives were based on the experiences reported and the fact that no studies on this subject had been found in the literature. 

The target audience (pregnant women, puerperal women, and family members) was thus defined, and it was determined that the website would be an important strategy for implementing efficient resources (thematic content and educational materials - booklets, videos, and apps) for home care of the newborn, enabling safe, quality care.

 The second stage, Design, establishes the learning objectives, focusing on the selection and planning of the educational content to be offered ^(^ 16 ^)^ . Therefore, the content selected, based on the authors’ previous experiences and research, concerns the process of discharge from the maternity hospital and care for newborns in the socio-family context, encompassing: the process of discharge from the maternity hospital; arrival at home; visits; sleep; bathing; umbilical stump care; diaper changes; skin care; breastfeeding; choking; transportation; vaccinations; general danger signs; and follow-up in health services, among others. 

The content was planned to be made available in sequential publications, on the Home tab, each publication will deal with a theme and with illustrations and easy-to-understand texts, as well as audios narrating each post. All the content was built based on the recommendations of the Brazilian Ministry of Health (MS), the Brazilian Society of Pediatrics (BSP), and the Brazilian Society of Immunizations (BSIM). All the content on the website was built jointly and collaboratively, with weekly meetings between the members of the group of authors. In this process, each participant took responsibility for the development of a predetermined number of publications, which promoted an effective distribution of tasks and also fostered a productive synergy, guaranteeing the cohesion and consistency of the material produced.

In addition, tabs were designed within the website that could contain the educational health technologies aimed at caring for newborns, produced and validated previously, as mentioned in stage 1: booklets, videos, and applications for mobile devices. These educational health technologies were scattered across the networks and could therefore be located at a single digital address, which could generate benefits for the target audience and the authors.

The following tabs were also planned: Virtual Library, containing hyperlinks to the sources used to build the website’s content; Frequently Asked Questions, covering questions and answers to the main doubts about newborn care; About Us, presenting the authors and their careers; and Contact Us, with contact addresses for the website’s authors/moderators. Also included in the planning were the Security and Privacy Policy, User Feedback, and Help Tutorial tabs, as well as an Interactive Chat.

In the third stage, Development, the conceptual aspects were considered: the way the content was presented, navigation, and the interface, as well as establishing the layout of the screens and the standard colors, establishing the website’s visual identity. In addition, a professional illustrator was hired to develop the illustrations for the thematic content, which were colorful and representative of the diversity of the Brazilian population.

The website was built by members of the project team using Wix, a simplified platform for creating, customizing, and administering websites, containing all the tabs planned in stage 3. In addition, the pages were built using Hypertext Transfer Protocol Secure (HTTPS), and optimized for viewing on mobile devices.

The fourth stage, Implementation, consisted of testing the navigation and reviewing the content of the website page. Using different types of technological resources (notebooks, computers, and smartphones), it was analyzed whether the tabs were working correctly, whether the videos were playing properly, whether the buttons and links were in the right direction, and whether the posts had the right content. Grammar, typing, coherence, and design were also checked. This testing phase was used to correct navigation errors, with a view to improving the target audience’s experience. After all this testing, the website was ready to be validated and evaluated, for subsequent dissemination to the target audience.

 In stage five, Evaluation, it is time to validate and evaluate the health education technology, which allows each stage to be reviewed the effectiveness of the technology to be analyzed, the content to be adapted and the users’ learning to be verified. In short, this stage allows the educational process to be improved, as it is able to correct deviations and establish the paths that are best suited to the learning objectives ^(^ 17 ^)^ . This technology was validated by experts and then, only after making the necessary adjustments, was it evaluated by the target audience. 

### Scenario

The study took place in a virtual environment.

### Time period

The website was built between February and March and the data was collected between April and May 2023.

### Population

The study population included expert participants (nurses and professionals in the field of Social Communication or Informatics) and the target audience (pregnant women, puerperal women, and family members of newborns).

### Selection criteria

To select and invite the experts, we consulted the Lattes Platform on the website of the National Council for Scientific Development (CNPq) to check that the possible participants met the following inclusion criteria: nurses with expertise in newborn care and/or expertise in pediatric nursing and/or previous experience in educational practices on home care, and professionals in the field of social communication or information technology with experience in websites. The exclusion criteria were as follows: professionals who only carried out administrative activities.

 In addition, to be included as an expert, it was necessary to achieve a minimum score of five points for the pre-selected criteria adapted from Fehring’s version ^(^ 18 ^)^ , whose score ranged from 01 to 05 points: Participation in a scientific event in the last two years on the subject of interest to the study - 01 point; Practice of at least five years in the area of interest to the study - 02 points; Publication in an indexed journal on the subject of interest to the study - 02 points; Specialist degree - 03 points; Master’s degree - 04 points; Doctoral degree - 05 points. To check the score, the data obtained from the Lattes Platform curriculum of each selected professional was also taken into account and those who did not reach the minimum score were excluded. 

To select the target audience, followers of the Instagram page of the extension project to which the authors belong were invited to take part, as well as other contacts from the research team itself. The inclusion criteria were: pregnant women, puerperal women, and family members of newborns who were over 18 and had access to the internet. Exclusion criteria were: pregnant women, puerperal women, and family members of newborns who had physical/mental limitations to answer the form and/or were illiterate.

### Sample definition

 Twenty experts and 20 individuals from the target audience took part in the study, as suggested by scientific evidence ^(^ 19 ^)^ , constituting a convenience sample. 

### Study variables

With regard to the experts, the following variables were collected: age, gender, professional training, professional qualifications, and length of time working in the study area. With regard to the target audience, the following variables were collected: age, gender, profession, level of education, whether they participate or will participate in the care of a newborn, kinship, or degree of relationship with the newborn. The other part of each instrument included questions specifically related to the object of the study, aimed at analyzing educational technology.

### Instruments used for data collection

The data collection process for validating and evaluating the website took place using Google Forms on the Google platform. The online survey page contained details of the project; the Free and Informed Consent Term (FICT) - which could be downloaded by the participant; a link to access the website; and an electronic form for data collection.

 For the experts, an adapted form was used based on a proposal for quality assessment criteria for health websites ^(^ 20 ^)^ , with questions divided into 11 groups, each containing a number of items (listed below in brackets) that dealt with the technical data and design of the website: accuracy (3); comprehensiveness (4); readability (7); credibility (8); advertising and sponsorship (1); security and privacy (4); ethical aspects (4); interactivity (5); user support (6); usability (11); and, accessibility (7). 

The items addressed specific issues within their groups: 1) Accuracy: scientific basis for the content, references and reliability of sources; 2) Comprehensiveness: offering information on care and preventive practices, their benefits and other sources for obtaining reliable information; 3) Readability: appropriate language, the purpose of the website and the way the content is presented; 4) Credibility: information on authorship, dates the website was created/updated and mention of the target audience; 5) Advertising and sponsorship: mention of support and partnerships; 6) Security and privacy: secrecy and encryption of personal data and security and privacy policy; 7) Ethical aspects: compliance with ethical and legal precepts and qualification of professionals and partnerships; 8) Interactivity: interactive tools, availability of an accredited and qualified moderator; 9) User support: frequently asked questions already answered; help tutorials, contacts made available, quick responses and evaluation of user satisfaction; 10) Usability: website navigation, design and search tool; 11) Accessibility: access on more than one device, quick loading of the website, availability of content in various formats, mechanisms for increasing the font size and accessibility bar.

 As for the target audience, an instrument used in an article on the construction and validation of educational videos ^(^ 18 ^)^ was used, having been adapted for the subject of the study in question. It contained six groups, each with a number of items (listed below in brackets): objectives (3); organization (6); website style (6); appearance (3); motivation (5); and usability (6). 

These items, within their specific groups, addressed issues such as: 1) Objectives: meeting the objective of providing guidance and help in the daily lives of families, and suitability for use by the target audience; 2) Organization: attractiveness, coherence, the way the content is presented and the importance of the topics covered; 3) Website style: accessibility of the vocabulary and clarity of the content; 4) Appearance: illustrations and presentation of the content; 5) Motivation: suitability for the profile of the target audience, logical sequence, subjects covered and encouragement of behavioral changes; 6) Usability: complexity of the website, integration of the tools, design, categories and loading of the website. In addition, each group had a space for suggestions/comments.

### Data collection

Potential participants were approached via WhatsApp or other social media, such as email and Instagram, so that they could take part in the process of validating or evaluating the website, without the use of lists that would allow their contact details to be identified or viewed by third parties. Participants could respond to the invitation and the survey within ten days. Because the sample was by convenience and the website was innovative and dynamic, all the experts and individuals in the target audience responded promptly to the material, without refusals or delays, which facilitated the data collection process.

### Data processing and analysis

 The data obtained from the collection instruments was exported from Google Forms to Microsoft Excel spreadsheet software and manually entered into tables according to each group of answers. The relevance of each item assessed was analyzed quantitatively, according to the different values: the total number of responses for totally disagree (1), partially disagree (2), partially agree (3), and totally agree (4). The Concordance Index (CI) was then calculated, due to its ability to measure the agreement between the participants’ answers, offering a quantitative metric. For this purpose, the score ranges from zero to one, calculated from the sum of the answers classified as three and four, divided by the total number of answers, with an index equal to or greater than 0.70 (70%) being considered a parameter of validity. As a result, items that did not meet this objective had to be corrected and/or modified, with the other items (CI > 0.7) being modified only if the suggestion was considered pertinent ^(^ 21 ^)^ . 

### Ethical aspects

All the ethical requirements set out in Resolution 466/12 of the National Health Council (CNS) were complied with. The study was approved by the Research Ethics Committee (CEP) of the Universidade Federal Fluminense, under opinion number 5.916.836 and Certificate of Submission for Ethical Appraisal (CAAE) 66091322.6.0000.5243. In addition, the Free and Informed Consent Term (FICT) was made available to the participants.

## Results

To build the health education website, the ADDIE model’s methodological stages of analysis, design, development, and implementation were completed. For the validation and evaluation of the website, the technology evaluation stage was completed, culminating in the results presented below.

The validation with the experts involved 18 (90%) professional nurses and two (10%) professionals in the field of Social Communication or Computer Science, 18 (90%) of whom were female and two (10%) male, aged between 25 and 69. As for the level of professional qualification, 12 (60%) had a doctorate, three (15%) had a master’s degree, four (20%) were specialists in the area of interest to the study and one (5%) had a degree in nursing.

Table 1 shows the experts’ assessment of accuracy, comprehensiveness, readability, credibility, advertising and sponsorship, security and privacy, ethical aspects, interactivity, user support, usability, and accessibility, using the CI by group and overall. 

**Table 1:** - Experts’ evaluation of the groups and items in terms of accuracy, scope, readability, credibility, advertising and sponsorship, security and privacy, ethical aspects, interactivity, user support, usability, and accessibility (n=20). Rio das Ostras, RJ, Brazil, 2023

**Groups and items**	**Strongly disagree/ Partially disagree**	**Strongly agree/ Partially agree**	**Index of Agreement**
**Accuracy**			
3 items	0	60	1
**Scope**			
4 items	2	78	0.97
**Readability**			
7 items	5	135	0.96
**Credibility**			
8 items	14	146	0.91
**Advertising and sponsorship**			
1 item	5	15	0,75
**Security and privacy**			
4 items	8	72	0.90
**Ethical aspects**			
4 items	7	73	0.91
**Interactivity**			
5 items	2	98	0.98
**User support**			
6 items	9	111	0.92
**Usability**			
11 items	10	210	0.95
**Accessibility**			
7 items	20	120	0.85
**Global Concordance Index**		**0.91**	

The average CI for all the items was greater than 0.7 (70%), and therefore the educational website was rated highly, with an overall average value of 0.91 (91%). Among the items evaluated, there was a variation between 0.75 (75%) and 1 (100%).

For the evaluation with the target audience, the following family members of the newborns took part: 12 (60%) mothers, two (10%) fathers, two (10%) cousins, two (10%) aunts, one (5%) brother and one (5%) grandfather, aged between 18 and 52. As for their level of education, seven (35%) had higher education, nine (45%) had secondary education and four (20%) had primary education. Of these, two (10%) said they were housewives, three (15%) were students, 14 (70%) were in various professions and one (5%) didn’t say.

Table 2 shows the target audience’s assessment of the objectives, organization, style of the website, appearance, motivation, and usability, by means of the CI per item and overall. 

**Table 2:** - Target audience’s assessment of objectives, organization, website style, appearance, motivation, and usability (n=20). Rio das Ostras, RJ, Brazil, 2023

**Groups and items**	**Strongly disagree/ Partially disagree**	**Strongly agree/ Partially agree**	**Index of Agreement**
**Objectives**			
3 items	0	60	1
**Organization**			
6 items	0	120	1
**Website style**			
6 items	0	120	1
**Appearance**			
3 items	0	60	1
**Motivation**			
5 items	0	100	1
**Usability**			
6 items	0	120	1
**Global Concordance Index**		**1**	

All the items got top marks, with an average CI of 1 (100%) and therefore greater than 0.7 (70%), showing that the educational website also got a satisfactory evaluation from the target audience, reaching an overall average value of 1 (100%).

Despite the satisfactory evaluation by both parties, the suggestions were analyzed and incorporated into the website’s adaptation as possible, with a view to ensuring the website’s further qualification. The experts’ suggestions that were taken on board concerned re-recording one of the audios, revising textual terms, adding a photo of the Child’s Handbook, and improving images, among others. Those from the target audience are concerned with textual adequacy.

**Figure 1:**
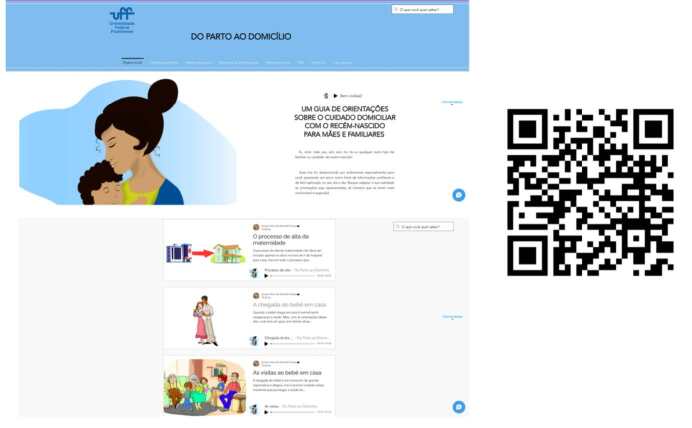
- Screenshot of the Home Page of the “ *Do Parto ao Domicílio* ” website and the respective Quick Response Code for access. Rio das Ostras, RJ, Brazil, 2023

 After this whole process, because of the good evaluation by the experts and the target audience, the content that had already been validated and evaluated was compiled into an educational booklet, which was added to the «Educational Booklets» tab. In addition, a number of comments praising the website were left, one of which concerned the brother of a newborn baby, who said he felt safer collaborating with his mother in-home care after learning about the website. This comment showed that the website is also capable of giving users confidence and an improved willingness to care. Figure 1 shows the layout of the website’s home page. 

## Discussion

 The health education website was designed, validated, and evaluated satisfactorily. The evaluation items reached excellent levels in relation to the established criteria. The validation by nursing, media, and IT professionals, with an overall Concordance Index of 0.91 (91%), shows that the website was considered suitable and could be used for an educational approach. Validation strategies for educational content in the health area are of the utmost importance in order to attribute reliability and validity to the materials used, especially given the great challenge faced today by the epidemic of false or controversial health news ^(^ 22 ^)^ . 

 Technology evaluation is also necessary, since it has an impact on the lives of its users, leading them to change their attitudes and behaviors, as well as enhancing care in favor of quality of life ^(^ 23 ^)^ . In this sense, in this study, the target audience’s evaluation obtained an overall Concordance Index equal to 1 (100%), demonstrating total satisfaction with the tool presented and a tendency towards subsequent frequent use of the website. 

 The number of scientific publications related to the creation of educational technologies has been increasing in recent years, given the various studies published in journals. In this regard, two studies have developed websites with educational objectives, one on cleft palate and the other on healthcare-related infection ^(^ 11 ^,^ 14 ^)^ . However, despite building new websites, these studies did not validate or evaluate them, unlike this study, which validated their content with experts and evaluated it with the target audience. 

 Another study, despite having built and validated its content with experts in the area of interest, has an important limitation: the lack of evaluation by the target audience ^(^ 22 ^)^ , which was carried out in this study, an essential factor for adapting usability and improving the provision of care for these individuals, as demonstrated by the comment left by one of the website evaluators in this study. 

 A study carried out in the United Kingdom evaluated the use of digital resources for self-help, diagnosis, and information-seeking among women going through the perinatal period. It showed that 41% of women use digital resources even to confirm information already provided by health professionals, with 70% of them using websites or apps to answer their questions, making it clear that online information retrieval and digital self-monitoring are increasingly integrated into self-care and offer opportunities to support the escalation of care and collaborate in decision-making ^(^ 24 ^)^ . It is therefore clear that websites can be used as a support and information network for the target audience, especially if they have been validated in advance by experts. 

 In addition, a study carried out in Slovakia looked at the creation of a website with educational content aimed at mothers of newborns and babies, family members, and the general public, and launched online advice on nursing care. The results showed that visitors were interested in the site and used it to answer their questions, especially about breastfeeding and nutrition, baby hygiene, and the care of sick babies, concluding that the provision of nursing care at a distance via the website effectively complemented the care provided in direct contact ^(^ 25 ^)^ . Similarly, the Brazilian website in this study allows users to interact and resolve doubts instantly, or within 24 hours, which was satisfactorily assessed by the participants in terms of objectives and user support. 

 With regard to the comprehension of educational material, the use of unnecessarily complicated language generally leads to ill-informed decision-making and a decrease in the individual’s interest in potentially beneficial health interventions, which becomes a barrier to the successful implementation of the intervention and can have a negative impact on health outcomes ^(^ 26 ^)^ . For this reason, it was decided to use simple and clear language, as well as images that described the texts presented, and audios that narrated all the content, in order to make the website more inclusive. In this sense, all the participants in the study, including experts and the target audience, felt that the vocabulary was accessible and appropriate for the intended audience and that the various functions were well integrated. 

 What’s more, digital platforms that provide information capable of contributing to the construction of knowledge are important resources for supporting learning, especially among young students ^(^ 27 ^)^ . Therefore, despite having a defined target audience, the website could also be of great value to students and health professionals, especially in the nursing field. In addition, ease of access, convenience, minimal costs, and the delivery of timely information are driving the increased use of digital media in health literacy, with websites standing out among the preferred resources ^(^ 28 ^)^ . 

 In addition, it is internationally recognized that families’ use of evidence-based newborn care practices in the home environment can save newborns’ lives. Thus, health education is an essential strategy in postnatal care programs to share with families the essential information and skills to provide quality and safe care to their newborns at home, thus promoting more favorable outcomes for babies ^(^ 1 ^)^ , which reinforces the importance of educational websites such as “ *Do Parto ao Domicílio* ” (“From Birth to Home”). 

As a limitation of this study, it is worth reflecting on the digital literacy presented by the participants, since most of them were individuals with access to the Internet and mobile devices and with a level of education between secondary and higher education, which may interfere with the results, demonstrating the need for new studies that include part of the population with other levels of education, both digital and educational.

However, this study makes a positive contribution to the advancement of scientific knowledge, providing a valuable contribution by building, validating, and evaluating a unique website that compiles up-to-date, diverse, and reliable information on home care for newborns, offering a centralized source of knowledge. Its availability not only promotes the expansion of digital health, accessible anywhere and at any time but also aims to mitigate inadequate practices, thus contributing to the reduction of neonatal and infant morbidity and mortality. In addition, this study fills a significant gap in the field of knowledge, meticulously presenting all the stages of a methodological study focused on websites related to the topic. In doing so, it not only offers valuable insights into home care for newborns but also presents a methodological standard that can guide future research in the area, strengthening the available evidence base.

## Conclusion

The educational website was built, validated, and evaluated satisfactorily since the evaluation items reached adequate levels in relation to the criteria by which it was evaluated. Therefore, it can be considered an appropriate tool for its purpose, as it brings benefits to the teaching-learning process for families regarding post-natal home care for newborns through its use. It can also be used to educate students and health professionals.

 The website is available for free and can be accessed at any time and from anywhere using laptops, computers, smartphones, or tablets at: https://www.dopartoaodomicilio.com.br/ . 
